# Cuproptosis-related lncRNA: Prediction of prognosis and subtype determination in clear cell renal cell carcinoma

**DOI:** 10.3389/fgene.2022.958547

**Published:** 2022-08-22

**Authors:** Youlong Huili, Shiwen Nie, Liguo Zhang, Anliang Yao, Jian Liu, Yong Wang, Lei Wang, Fenghong Cao

**Affiliations:** Department of Urology, North China University of Science and Technology Affiliated Hospital, Tangshan, Hebei, China

**Keywords:** cuproptosis, ccRCC, lncRNA, prognosis, subtype

## Abstract

**Background:** Clear cell renal cell carcinoma (ccRCC) is the most common type of renal cell carcinoma, accounting for approximately 70% of all RCC cases. Cuproptosis, a novel mechanism of cell death, may be a potential target for intervention in tumor development.

**Methods:** Cuproptosis-related prognostic lncRNAs were identified by co-expression analysis and univariable Cox regression. Five lncRNA profiles were obtained by LASSO regression analysis, and a model with high accuracy was constructed to assess the prognosis of ccRCC patients based on these cuproptosis-related lncRNAs. Survival analysis and time-dependent ROC curves were performed for the *α* and *β* groups, and the results confirmed the high accuracy of the model in predicting the prognosis of ccRCC patients. Immunoassay, principal component analysis (PCA), and drug sensitivity analysis were also performed for different risk categories. Finally, we classified ccRCC patients into two different subtypes by consistent class clustering, and performed immune checkpoint activation, tumor microenvironment analysis, PCA, and drug sensitivity analysis for different subtypes.

**Results:** We developed a prognostic model using five cuproptosis-associated lncRNAs, which was found to be highly accurate in predicting ccRCC patients’ prognosis. Immunotherapy may be more beneficial to the hyper-risk category and the C2 subtype.

**Conclusion:** The results of this study confirm that five cuproptosis-associated lncRNAs can be used as potential prognostic markers for ccRCC.

## Introduction

Renal cell carcinoma (RCC) is one of the three major tumors of the urinary system, with approximately 4,00,000 new cases and 1,70,000 cancer-related deaths reported in 2018, according to global cancer statistics (2020). Clear cell renal cell carcinoma (ccRCC) is the most prevalent pathological type, comprising approximately 70% of all RCC cases (Shuch et al., 2015). Despite the fact that surgical resection is presently the primary therapeutic option for ccRCC, research has revealed that 30%–40% of patients with localized lesions will experience recurrence following surgery (Choueiri and Motzer 2017). Immunotherapy has grown in importance for the cancer treatment paradigm in recent decades and been acknowledged as a promising therapeutic option, (Hoos 2016; Aoun et al., 2017; Kamal et al., 2019). However, in the vast majority of malignancies, only one-third of patients react to immune checkpoint inhibitors (Tang et al., 2020). In the treatment of RCC, the effectiveness of Sorafenib and Sunitinib is clinically well documented. (Nassif et al., 2017; Tatsugami et al., 2018).However, in some cases, the efficacy of treatment is greatly compromised by the development of resistance to Sorafenib and Sunitinib.(Qu et al., 2016; Ishibashi et al., 2018). Therefore, it is essential to investigate how to enhance immunotherapy for ccRCC and to select the appropriate treatment regimen based on the relevant subtype.

Autophagy death, apoptosis, necroptosis, and other cell death pathways have all been found necessary for the elimination of damaged and redundant cells from the body (Tsvetkov et al., 2022). Copper’s role in disease has been studied since the 1990s. In 1991, Pocino et al. found that a high intake of copper slows down both cellular and humoral immune responses (Pocino et al., 1991). In the early 21st century, Linder (2012) discovered that continuous exposure of cells and tissues to excess copper could activate p53-dependent or independent pathways to trigger “programmed cell death” or “apoptosis.” Copper-induced “apoptosis” has been demonstrated in hepatocytes, splenocytes, and thymocytes in later research (Mitra et al., 2012; Keswani et al., 2015). Tsvetkov et al. (2022) have discovered a new type of cell death called “cuproptosis,” which is triggered by the aggregation of mitochondrial lipid acylated proteins, and the instability of Fe-S cluster proteins, resulting in cell death. This research elucidates the biology of inherited copper overload illnesses, and provides a novel approach to cancer treatment based on copper toxicity (Kahlson and Dixon 2022). As a result, this novel cell death signaling pathway could be a viable intervention target in tumorigenesis, suggesting a new anti-tumor strategy.

Recent research indicates that long-stranded noncoding RNAs (lncRNA) are involved in a variety of tumor progression mechanisms, including carcinogenesis, proliferation, migration, invasion, metastasis, and angiogenesis (Tan et al., 2022). Nonetheless, no research has been conducted on the prognostic model of cuproptosis-related lncRNAs. So, the goal of this study was to create a prognostic model of cuproptosis-related lncRNA to assess and improve the prognosis of ccRCC, as well as to study the differences in the tumor microenvironment and related subtypes to provide more evidence for the individualized treatment of ccRCC.

## Materials and methods

### Information acquisition from patients with ccRCC

We extracted mRNA sequencing (RNA-seq) data and accompanying clinical information for ccRCC patients from the TCGA database (https://portal.gdc.cancer.gov/) in April 2022. The dataset included 539 ccRCC tissue samples and 72 adjacent normal tissue samples, but data from seven ccRCC patients were excluded because they did not have complete clinical data. All 530 patients with ccRCC were randomly assigned into *α* and *β* groups, in a 1:1 ratio, using Strawberry Perl and the caret R program. As an independent validation cohort, the E-MTAB-1980 cohort from the EMBL-EBI database (https://www.ebi.ac.uk/) was used (Sato et al., 2013). The clinical data for the above two cohorts are shown in Supplementary Table S1.

### Selection of cuproptosis-related genes and related lncRNAs

We identified 13 cuproptosis-associated genes through a study of relevant literature (Tsvetkov et al., 2022), and extracted 4,438 lncRNAs from TCGA-KIRC after screening the synthetic data matrix by Strawberry Perl and limma R packages and identified 180 cuproptosis-related lncRNAs by correlation analysis (|correlation coefficient| > 0.4, *p* < 0.001). We subsequently obtained 73 cuproptosis-related differentially expressed lncRNAs by differential analysis (|Log 2 FC| > 1 and *p* < 0.05).

### Model construction validation

Univariable Cox analysis was performed to extract 18 OS-related lncRNAs from the 73 cuproptosis-related lncRNAs listed above (*p* < 0.05), then LASSO regression for the *α* group, to develop a model comprising five cuproptosis-related lncRNAs. The risk score is derived using the formula below (h0(t) = 0.998823001):

$$\mathrm{risk}\;\mathrm{score}=\mathrm{EXP}\left[\sum_{k=1}^{n}\mathrm{coef}\left(\mathrm{IncRN}A^{k}\right)*\exp\left(\mathrm{IncRN}A^{k}\right)\right]*h_{0}\left(t\right)$$

The *α* group, *β* group, all patients group, and E-MTAB-1980 cohort were then divided into a hyper-risk category (above the median) and a hypo-risk category (below the median), based on the median risk score of the *α* group. Finally, survival analyses and time-dependent (ROC) curves were performed separately for the *α* group, *β* group, all patients group, and E-MTAB-1980 cohort to test the accuracy of the model and to calculate the distribution of high- and low-risk patients among all patients.

### Independent prognostic analysis, clinical correlation analysis, and stratified analysis

To assess if the model provides an independent prognostic indicator, univariable and multivariable prognostic analyses of risk scores were conducted. In addition, assessments of several clinical strata were performed, to assess the predictive power of each stratum (Hidalgo and Goodman 2013).

### Nomogram construction and calibration

To enhance the model’s validity and precision, we developed a prediction nomogram based on patient clinicopathological parameters (pathological stage and age) and risk scores. Each variable is assigned a score in the nomogram, and the final score for each sample is the sum of these variables values (Iasonos et al., 2008). Calibration curves describe the projected values between the predicted 1-year, 3-years, and 5-years survival real events and the predicted outcomes.

### Immune infiltration, immune checkpoint enrichment analysis, and prediction of clinical treatment

Using six distinct platforms of analysis, including TIMER, CIBERSORT, XCELL, QUANTISEQ, MCPCOUNTER, EPIC, and CIBERSORT, we compared the variance of immune cell infiltration between high and hypo-risk categorys. In addition, to gain a better understanding of the immune milieu in various risk categories, we evaluated TME scores and immune checkpoint activation between risk categories, using the ggpubr R program. To better comprehend the function of the model in clinical drug therapy, we evaluated patient reaction to treatment using the R package pRRophetic.

### Determination of different subtypes

To explore the response of ccRCC to immunotherapy, molecular subgroups were defined by examining five cuproptosis-associated lncRNAs in a construct model, and survival study was conducted for each subtype. To explore the immune infiltration characteristics of various subtypes, the ESTIMATE method was used to calculate the tumor microenvironment characteristics of various subtypes. In addition, we evaluated the differences in common immune checkpoint expression between subtypes. We evaluated the semi-inhibitory concentration (IC50) values of chemotherapeutic medicines regularly used to treat ccRCC, in order to determine drug sensitivity for distinct subtypes.

### Statistical analysis

Statistical analysis was performed in this study using R4.1.2. Pearson correlation analysis was used to analyze the correlation between cuproptosis-related genes and cuproptosis-related lncRNAs. Student’s *t*-test was used to determine the differences in expression of cuproptosis-related lncRNAs between tumor and normal tissues, while chi-square test was used to compare the differences in proportions. Kaplan-Meier analysis and the log-rank test were used to compare OS and DFS between subgroups. univariate and multivariate Cox regression analyses were performed to determine independent predictors of OS. Immune cell infiltration, immune checkpoints, and TME scores were compared between high- and low-risk groups and between subtypes using the Wilcoxon test. Spearman correlation analysis was used to explore the correlation between the degree of immune cell infiltration and risk scores across platforms, between drug sensitivity and subtypes, and between drug sensitivity and risk groups. All statistical tests were two-tailed and *p* < 0.05 was considered statistically significant and labeled with * *p*-value < 0.05, ** *p*-value ≤ 0.01, *** *p*-value ≤ 0.001 and ** ** *p*-value ≤ 0.0001.

## Results

### Identification of cuproptosis-related lncRNAs

**FIGURE 1:**
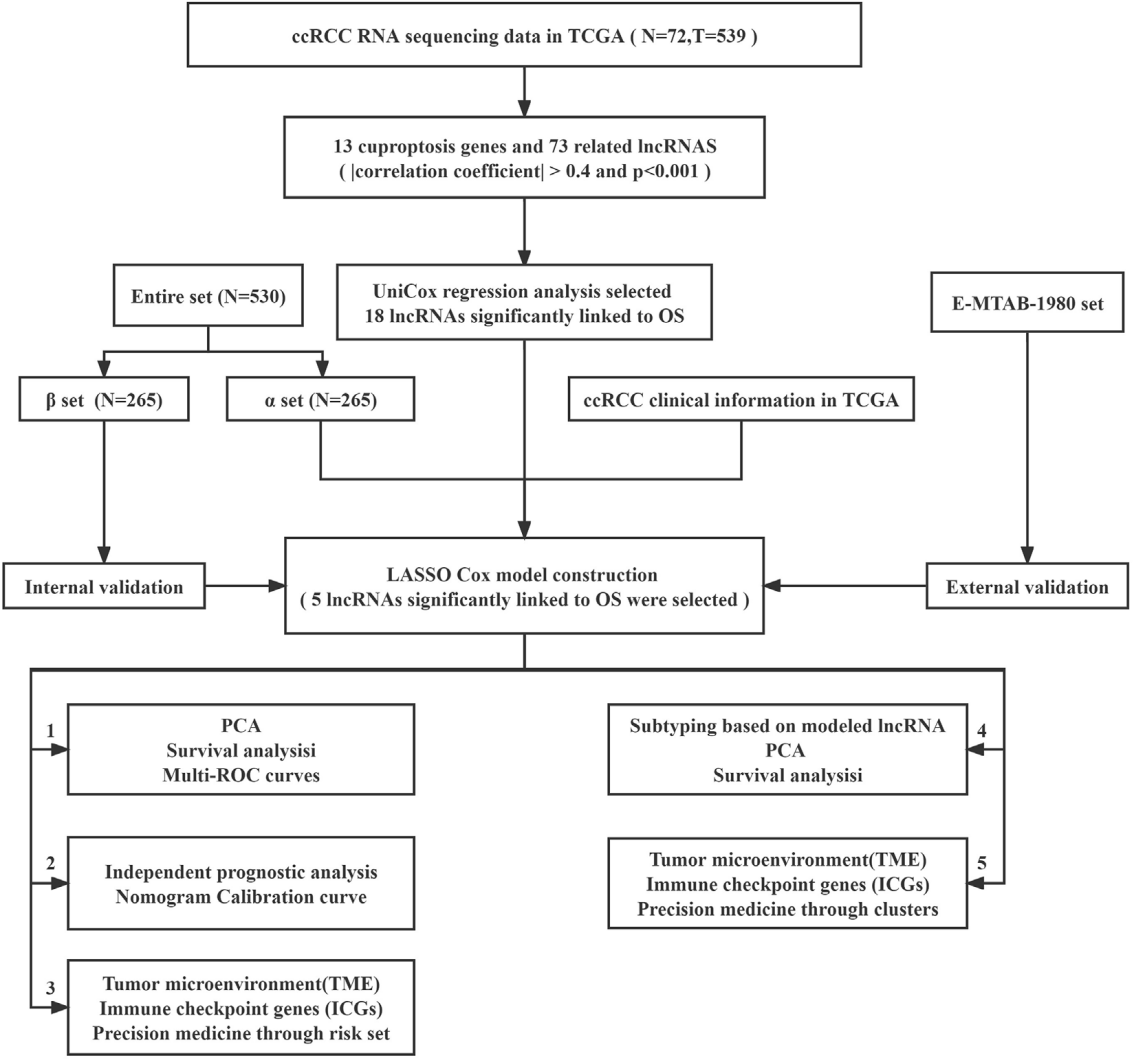
Flow chart of this study.

**FIGURE 2:**
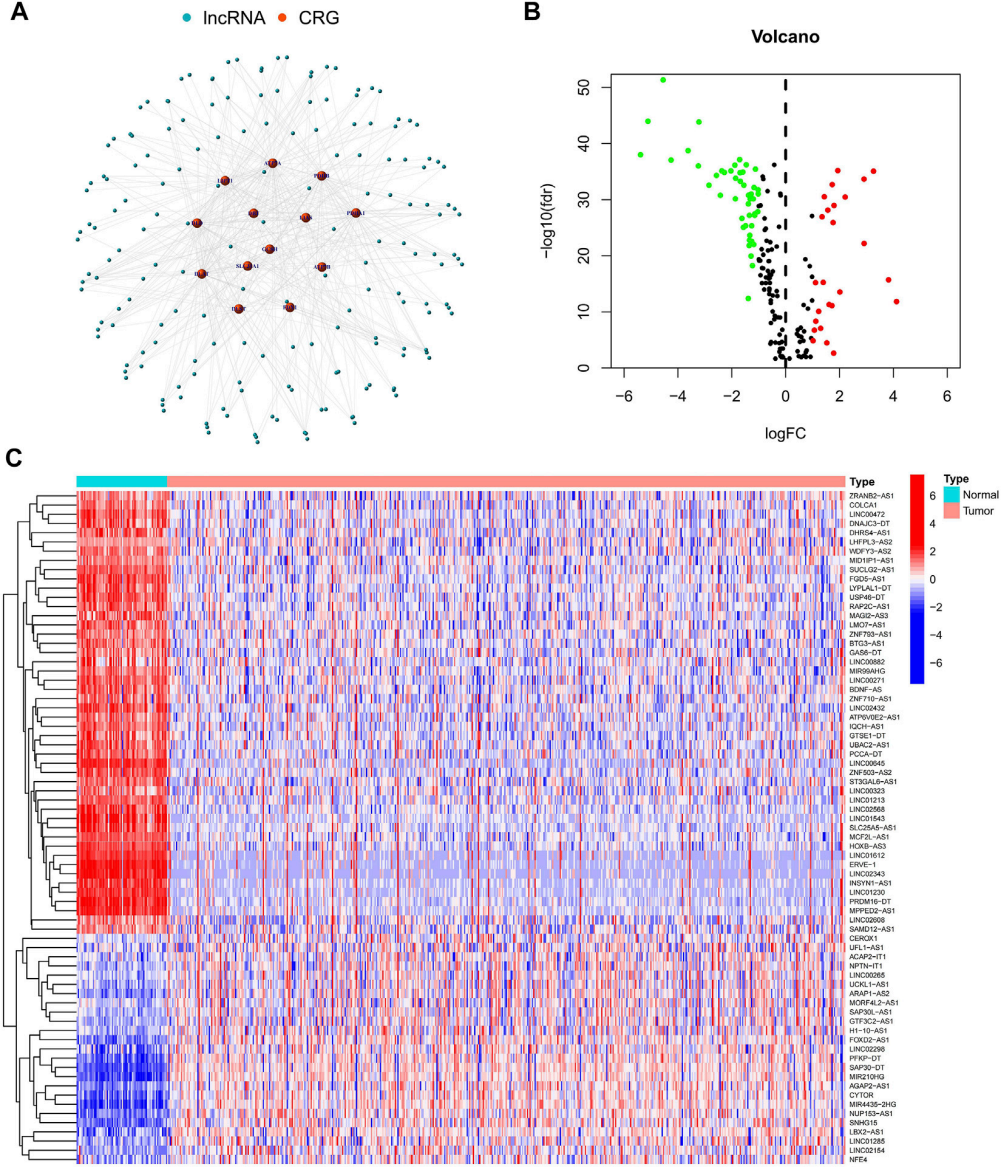
Identification of cuproptosis-related lncRNAs. **(A)** Network map of cuproptosis-related genes and lncRNAs; **(B)** Volcano map of 73 differentially expressed cuproptosis-related lncRNAs; **(C)** Heat map of 73 differentially expressed cuproptosis-related lncRNAs.

The study’s flowchart is depicted in Figure 1. The Cancer Genetic Atlas (TCGA) was used to retrieve mRNA sequencing (RNA-seq) data and accompanying clinical information for 530 ccRCC patients. The collection included 539 samples of tumor tissue and 72 samples of surrounding normal tissue. After synthesizing the data matrix by Strawberry Perl and limma R package screening, 4,438 lncRNAs were extracted from TCGA-KIRC (Supplementary Table S2). To understand the relationship between the above lncRNAs and cuproptosis-related genes, we set the |correlation coefficient| as 0.4 and *p* < 0.001 to obtain 180 cuproptosis-related lncRNAs and plotted The relationship network of the two was plotted (Figure 2A; Supplementary Table S3). By analyzing the differential expression profiles of 180 lncRNAs between normal and tumor tissue samples (Supplementary Table S4), we obtained 73 differentially expressed copper death-associated lncRNAs (|Log 2 FC| > 1 and *p* < 0.05). We found that 34% (25 out of 73) were in an upregulated state, while the others were in a downregulated state (Figure 2B; Supplementary Table S5). Figure 2C shows more clearly the difference in expression of the above 73 cuproptosis-related lncRNAs between tumor tissues and normal tissues.

### Creation of a predictive model comprising five cuproptosis-related lncRNA characteristics

**FIGURE 3:**
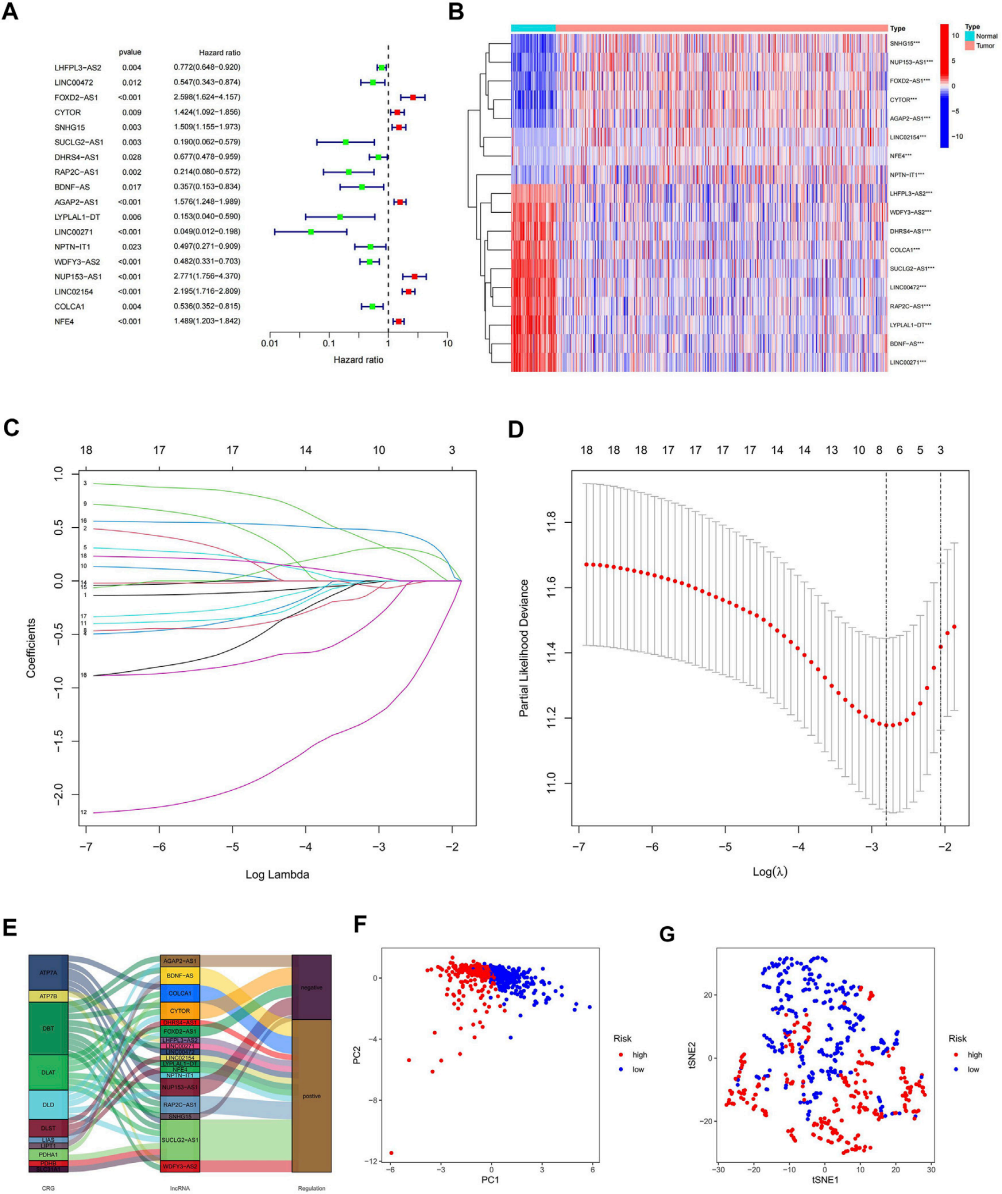
Creation of a predictive model comprising five cuproptosis-related lncRNA characteristics. **(A)** Prognostic value of 18 cuproptosis-related lncRNAs; **(B)** Prognostic value of 18 heat maps of cuproptosis-related lncRNAs; **(C)** Expression coefficient maps of LASSO regression; **(D)** Cross-test maps of penalty terms; **(E)** Sankey maps of regulatory relationships between cuproptosis-related genes and lncRNAs; **(F)** PCA maps of high- and hypo-risk categories; **(G)** t-NES validation maps of high- and hypo-risk categories.

To further understand the relationship between the above 73 cuproptosis-related lncRNAs and the prognosis of ccRCC patients, we performed univariable Cox regression analysis. Eighteen cuproptosis-related lncRNAs were significantly associated with overall survival (OS) of ccRCC patients (*p* < 0.05), and heat maps were drawn for them (Figures 3A,B). To avoid overfitting the prognostic features, we first randomized 530 patients in the TCGA-KIRC cohort 1:1, into *α* (*n* = 265) and *β* groups (*n* = 265) using the “caret. package” in R software. We then performed LASSO regression analysis on the above 18 cuproptosis-related lncRNAs (Figures 3C,D), and finally extracted five cuproptosis-related lncRNAs (FOXD2-AS1, SUCLG2-AS1, LINC00271, NUP153-AS1, LINC02154) in ccRCC (Supplementary Table S6). According to the Sankey diagram, FOXD2-AS1 and NUP153-AS1 were negatively controlled by cuproptosis-related genes, but the other three lncRNAs are positively regulated (Figure 3E). The risk score for each ccRCC patient was then calculated using the five lncRNAs listed above: risk score = EXP [(0.558190947296863*FOXD2-AS1) + (−1.20602359077768*SUCLG2-AS1) + (−2.26877398974126*LINC00271) + (0.437610996540462*NUP153-AS1) + (0.590181559841534*LINC02154)]*0.998823001. Finally, we classified the patients in group *α*, group *β*, and all groups into hyper-risk and hypo-risk categories, based on the median risk score of group *α*, respectively. The PCA findings demonstrated that distinct PCs were created between the high and hypo-risk categories (Figure 3F), and the t-SNE confirmed that the two groups could be discriminated with precision (Figure 3G).

### Validation of a predictive model based on cuproptosis-related lncRNA

**FIGURE 4:**
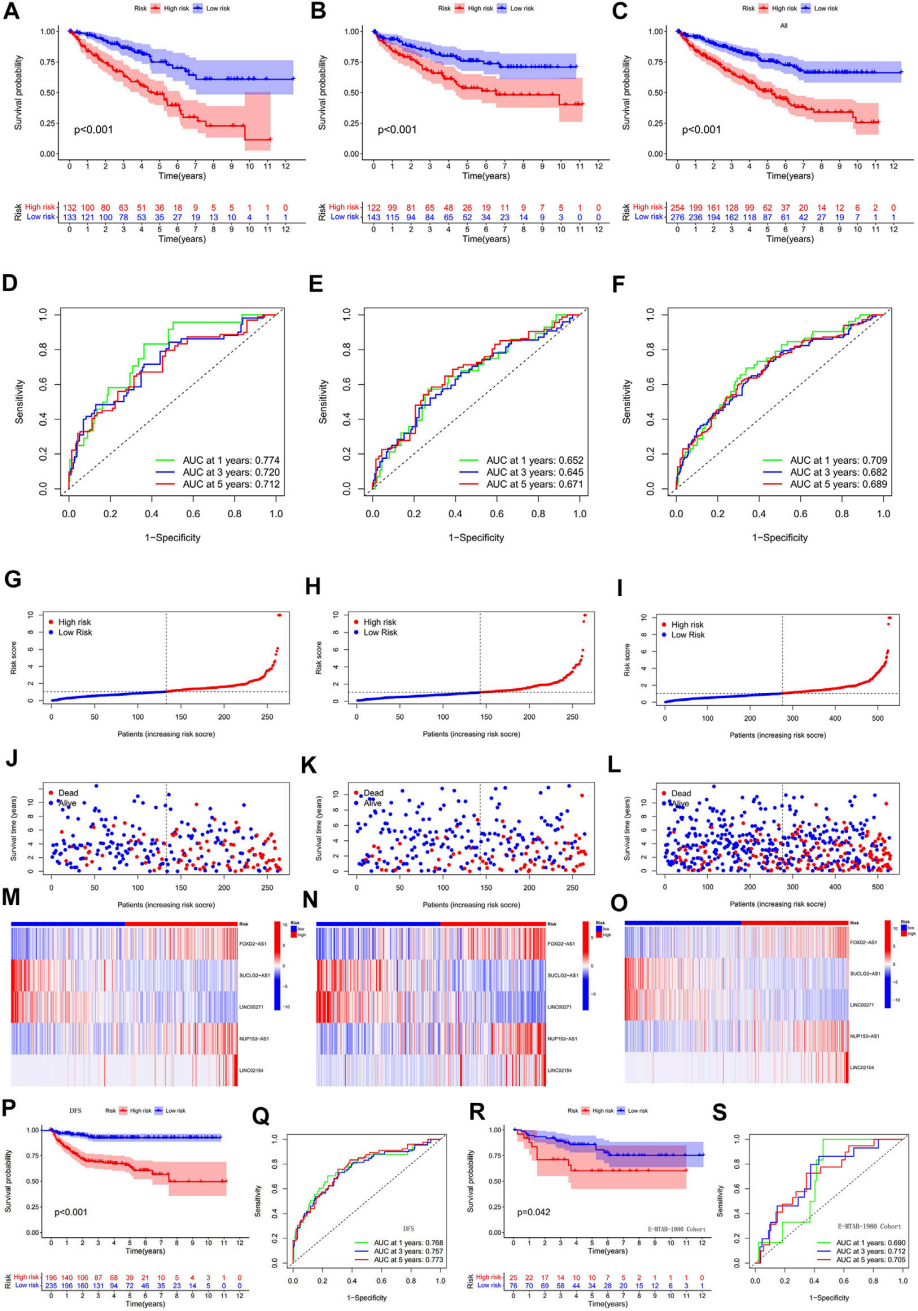
Validation of a predictive model based on cuproptosis-related lncRNA. **(A)** survival analysis of *α* group; **(B)** survival analysis of *β* group; **(C)** survival analysis of all groups; **(D)** ROC curves of *α* group; **(E)** ROC curves of *β* group; **(F)** ROC curves of all groups; **(G–O)** risk score distribution, survival status, survival time and related expression of *α*, *β* and all groups relationship plots; **(P)** survival analysis of 431 patients with DFS; **(Q)** ROC curves of 431 patients with DFS; **(R)** survival analysis of the E-MTAB-1980 cohort; **(S)** ROC curves of the E-MTAB-1980 cohort.

To verify the validity and accuracy of the prognostic model, we performed survival analysis for the *α* group, the *β* group, and a cohort containing all patients. The results of all three cohorts showed a poor prognosis in the hyper-risk category compared with the hypo-risk category (*p* < 0.01; Figures 4A–C), and the relevant clinical characteristics, except that for G1–2 classification, M1, and N1 staging. This supported the idea of a poor prognosis in the hyper-risk category (Supplementary Figure S1). By displaying time-dependent ROC curves, we determined 1-year, 3-years, and 5-years survival AUC values of 0.774, 0.720, and 0.712 for the *α* group (Figure 4D). The AUC values for the *β* group and all other groups exceeded 0.64. This suggests that the model has some significance in predicting the prognosis of ccRCC patients (Figures 4E,F). By correlating the distribution of risk scores, survival status, survival time, and related expression in the aforementioned three groups, it is evident that the number of deaths in ccRCC patients increased with increasing risk scores, and that FOXD2-AS1, LINC02154, and NUP153-AS1 were enriched in the hyper-risk category, whereas the other two cuproptosis-related lncRNAs were enriched in the hypo-risk category (Figures 4G–O). Assessing DFS (disease-free survival) in oncology studies has an important position. We compiled clinical data from 530 patients in the TCGA-KIRC cohort, to obtain the DFS of 431 patients, performed survival analysis, and plotted ROC curves for them. It was easy to find that the DFS of the high and low risk groups were significantly different, and the prognosis of the low risk group was still higher than that of the high risk group (Figure 4P). In addition, the area under the curve (AUC) values of the ROC curves were all greater than 0.7 (Figure 4Q). Since the model was constructed based on a public dataset with weak persuasive power, we obtained an external dataset, the E-MTAB-1980 cohort, for validation. Survival analysis of the E-MTAB-1980 cohort showed that the low-risk group had a good prognosis (*p* < 0.05, Figure 4R) and the ROC curves for this cohort had 1-year, 3-years and 5-years AUC values of 0.69, 0.712, and 0.705 (Figure 4S). Validation of the E-MTAB-1980 cohort also demonstrated the potential significance of the model in predicting ccRCC prognosis.

### Risk score as an independent prognostic factor and the construction of the nomogram

**FIGURE 5:**
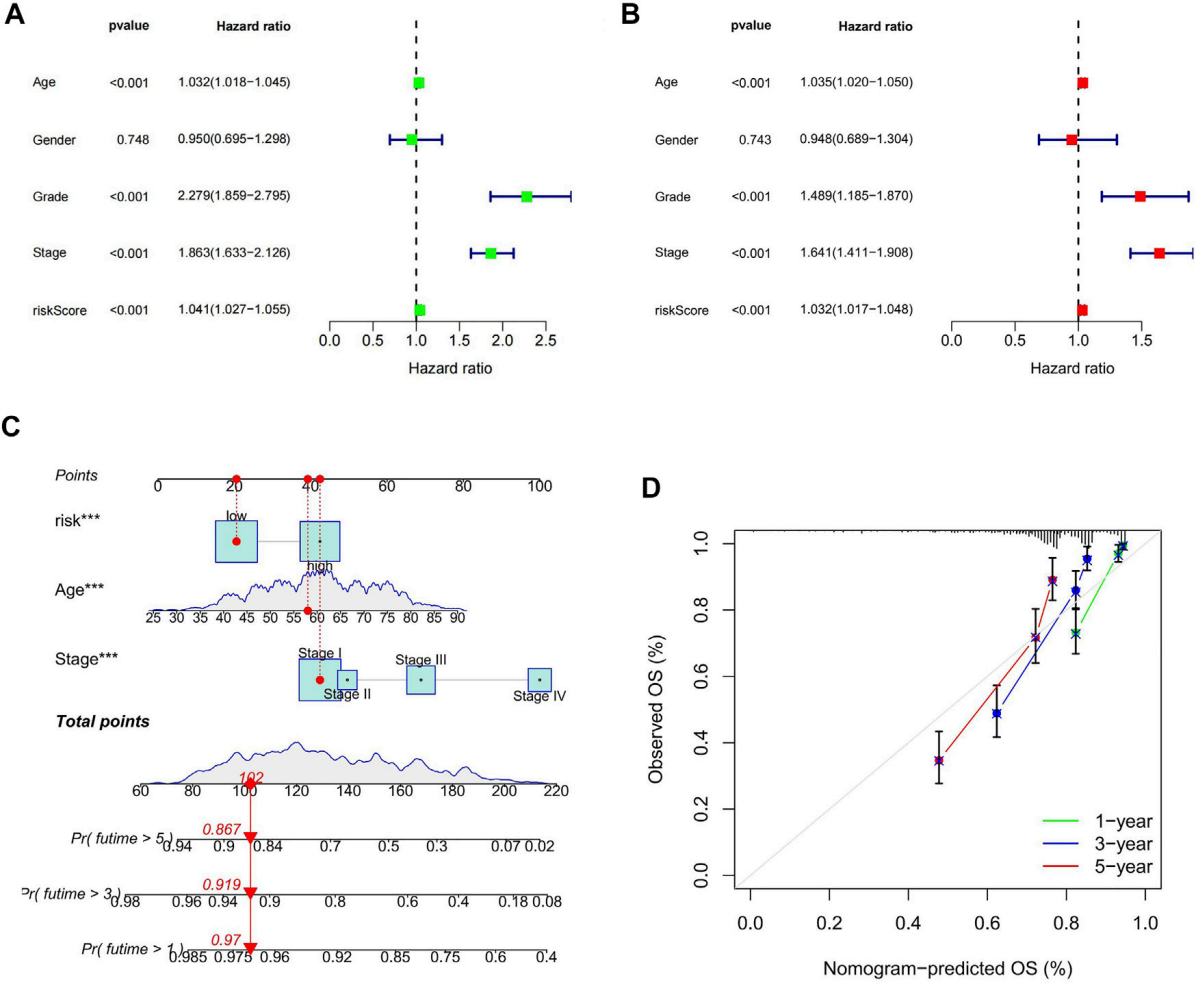
Risk score as an independent prognostic factor and the construction of the nomogram. **(A)** results of one-way Cox regression analysis; **(B)** results of multi-way Cox regression analysis; **(C)** Nomogram predicting prognosis of ccRCC patients; **(D)** calibration curve.

The outcomes of uni- and multi-variate Cox regression analyses indicated that the cuproptosis-related lncRNA model’s risk score was an independent predictor of prognosis in ccRCC patients (*p* < 0.05; Figures 5A,B). We validated once again that the risk score was an independent predictor of prognosis in ccRCC patients by analyzing the combined data from the *β* group and the E-MTAB-1980 cohort (*p* < 0.05; Supplementary Figure S2). We found that grading, staging and risk score were all independent prognostic factors, for which we compared the AUC values of 1, 4, 6, and 8 years for clinicopathological characteristics and risk score. We found that the AUC values at 1, 4, and 6 years for tumor stage and grading were higher than those for risk score, and notably the AUC at 8 years was highest for risk score. Although the AUC values of tumor staging and grading at 1, 4, and 6 years were significantly higher than those of risk fraction, the overall AUC values of risk fraction were higher, indicating that the model has some significance in predicting the prognosis of ccRCC (Supplementary Figure S3). To improve the validity and accuracy of the model, a nomogram was developed by combining risk score, age, and tumor stage, to predict the 1-year, 3-years, and 5-years recurrence-free survival (RFS) of patients with ccRCC (Figure 5C). The calibration curves demonstrated a high degree of concordance between the actual results and those predicted by the nomogram (Figure 5D).

### The hyper-risk category had higher immune activity and was more drug sensitive

**FIGURE 6:**
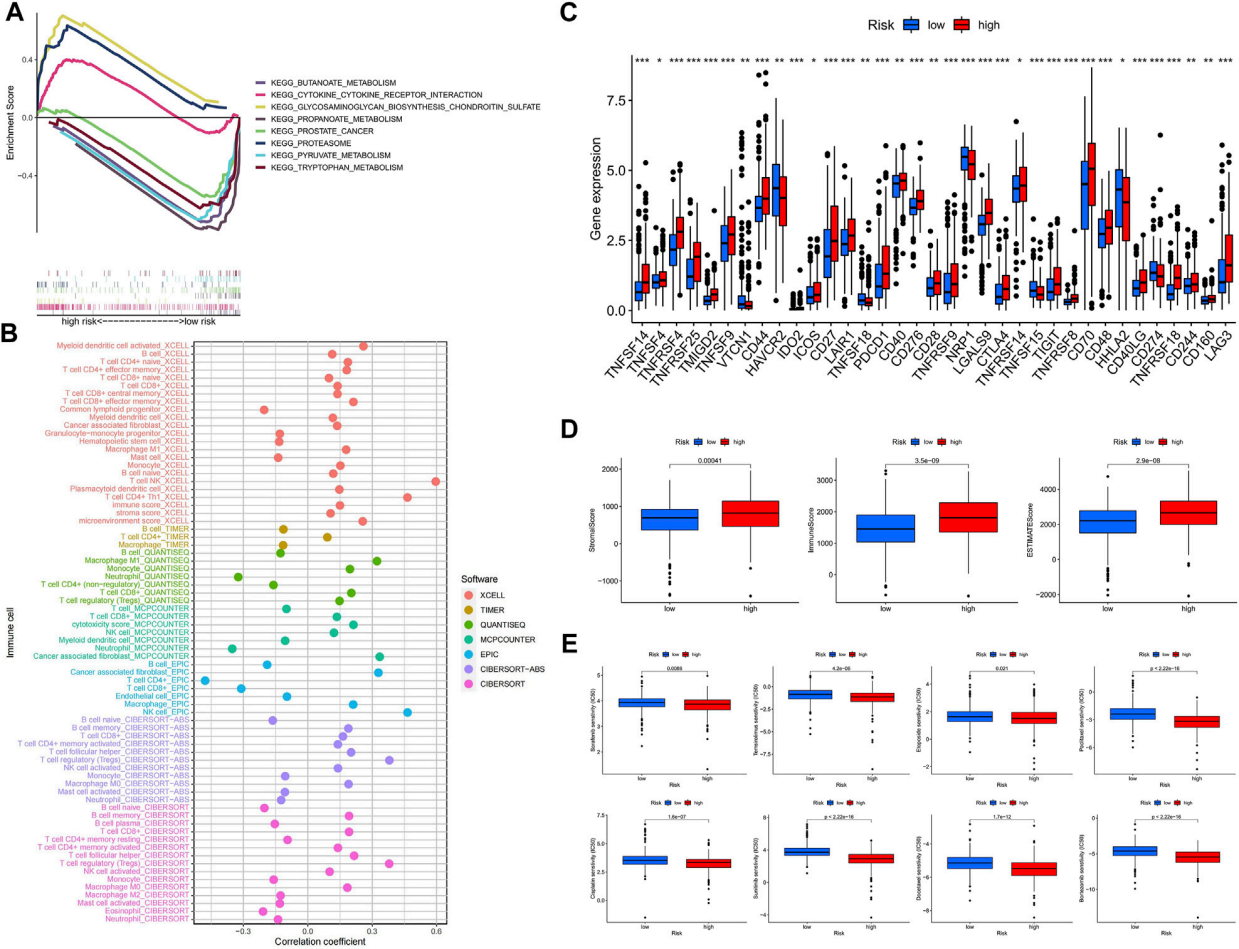
The hyper-risk category had higher immune activity and was more drug sensitive. **(A)** GSEA analysis of high and hypo-risk categories; **(B)** bubble plots of correlation analysis between immune cells and risk scores for different platforms; **(C)** correlation analysis between high and hypo-risk categories and immune checkpoints; **(D)** TME scores for high and hypo-risk categories; **(E)** prediction of immunotherapy efficacy for high and hypo-risk categories.

Using GSEA software, we looked at the KEGG pathway enrichment between high and hypo-risk categories, and discovered that immune-related pathways were enriched in the hyper-risk category (Figure 6A). To better understand immune cell infiltration in high and hypo-risk categories, we plotted immune cell bubble maps using algorithms from various platforms. The results revealed that the majority of immune cells, including T cell CD8^+^ naïve, cancer associated fibroblasts, B cells, and regulatory T cells (Tregs), were positively correlated with risk scores (Figure 6B). This indicates that the hyper-risk category exhibited a greater degree of immune cell infiltration. The correlation between the 47 immune checkpoints and the high- and hypo-risk categories was then investigated. We determined that the majority of immune checkpoint-related genes were significantly different between the high- and hypo-risk categories, and overwhelmingly upregulated in the hyper-risk category (Figure 6C). This means that immunosuppressive therapy for ccRCC patients can be tailored to the appropriate immune checkpoint inhibitor, based on their risk category. Using the ESTIMATE package, we then evaluated the tumor microenvironment (TME) scores in the high- and hypo-risk categories. Higher stromal or immune scores indicated a greater relative content of stromal or immune cells in the TME, whereas estimated scores indicated the accumulation of stromal and immune scores in the TME. The results revealed that hyper-risk patients had higher stromal, immune, and estimated scores than hypo-risk patients (Figure 6D). In conclusion, hyper-risk patients with ccRCC had higher levels of immune infiltration, suggesting that immunotherapy may be more beneficial for these patients. Docetaxel, bortezomib, sorafenib, temsirolimus, etoposide, paclitaxel, cisplatin, and sunitinib had significantly lower IC50 values in the hyper-risk category compared to the hypo-risk category (Figure 6E).

### Confirmation of different subtypes

**FIGURE 7:**
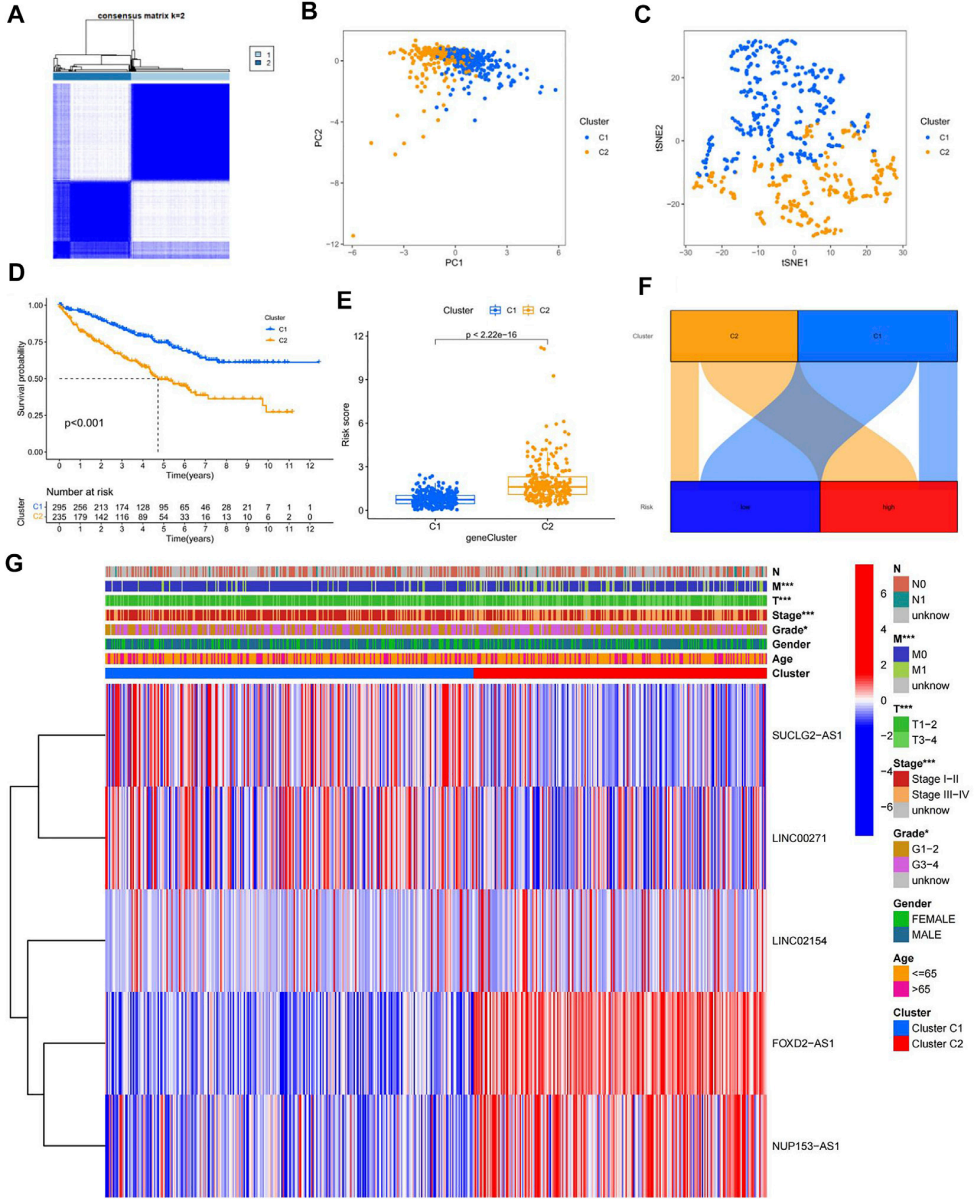
Confirmation of different subtypes. **(A)** results of the consistent clustering algorithm at *k* = 2; **(B)** PCA plots for different subtypes; **(C)** t-NES validation plots for different subtypes; **(D)** K-M curves for different subtypes; **(E)**box plot of subtypes versus risk scores; **(F)** Sankey plots of high and low risk groups versus different subtypes; **(G)** Heat map of correlation between clinicopathological features and subtypes.

To better investigate individualized precision therapy, we used the ConsensusClusterPlus (CC) R software package, to stratify patients with ccRCC into two subtypes, based on the five cuproptosis-related lncRNA expressions described previously (Figure 7A; Supplementary Figure S4). PCA results showed that different PCs formed between the two subtype groups (Figure 7B), and t-SNE confirmed that the two groups could be distinguished (Figure 7C). Kaplan-Meier analysis curves clearly showed that OS was considerably higher in patients with the C1 subtype than in those with the C2 subtype (Figure 7D; *p* < 0.001). By analyzing the distribution of risk scores between the two subtypes, we plotted the associated box plots. There was a significant difference in the distribution of risk sores between the two subtypes, and the risk scores were higher for subtype C2 than for subtype C1 (Figure 7E). The Sankey plots of the high- and low-risk groups with the two subtypes also clearly showed that patients in the high-risk group had a high overlap with subtype C2 and patients in the low-risk group with subtype C1 (Figure 7F). To investigate the correlation between different subtypes and clinicopathological features, we analyzed and plotted the correlation heat map. The results showed that T, M, stage, grade and subtype of the tumor were significantly correlated, while N, age and gender were not (Figure 7G).

### Subtype C2 is more susceptible to immunotherapy

**FIGURE 8:**
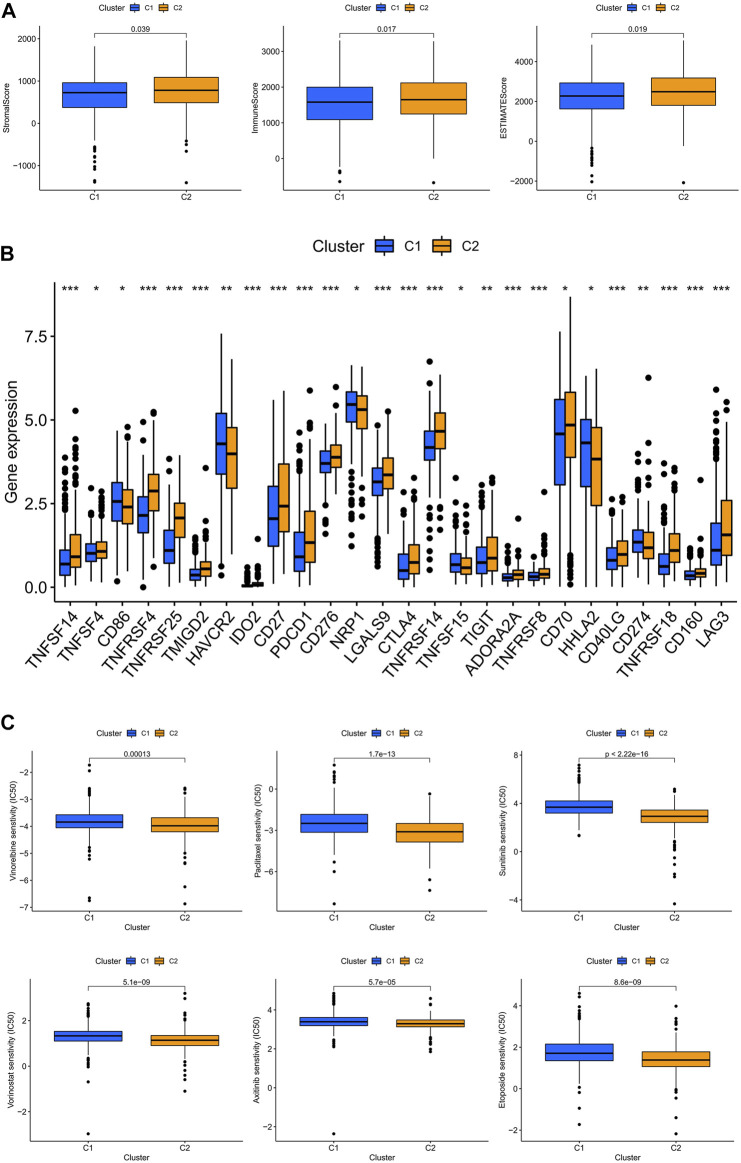
Subtype C2 is more susceptible to immunotherapy. **(A)** TME scores for both subtypes; **(B)** immune checkpoint correlation analysis for both subtypes; **(C)** immunotherapy prediction for both subtypes.

The TME scores of the C2 subtype were significantly higher than those of the C1 subtype, indicating that there are significant differences in the tumor microenvironment between the two subtypes, and that the C2 subtype may be more responsive to immunotherapy (Figure 8A). The results showed that the majority of immune checkpoints were differentially expressed between the two subtypes, and 21 immune checkpoints were upregulated in the C2 subtype, except CD274, CDE86, HAVCR2, NRP1, and HHLA2 (Figure 8B). By drug sensitivity analysis, we discovered that the IC50 of vinorelbine, paclitaxel, sunitinib, vorinostat, axitinib, and etoposide was significantly lower for the drug subtype C2 than for drug subtype C1, indicating that drug subtype C2 is more susceptible to immunotherapy. This provided an important clue for the individualized precision medicine treatment of patients with ccRCC (Figure 8C).

## Discussion

Copper, an essential mineral element for all living organisms, plays a crucial role in numerous biological processes, including mitochondrial respiration, iron absorption, and antioxidation. According to a recent study, copper ion functions as a double-edged sword because it is an essential enzymatic cofactor in life processes, but its excessive concentration causes proteotoxic stress, by binding to lipoic acid-associated enzymes of the tricarboxylic acid cycle, resulting in cell death. Tsvetkov et al. (2022) coined the term cuproptosis for this unique form of cell death, which is distinct from any other cell death mechanism previously discovered. As widely distributed in various fluids in the human body, lncRNA has an important position in various pathophysiological processes such as human disease screening (Yuan et al., 2020). As early as 2006, lncRNA PCA3 in urine was proposed as a biomarker for prostate cancer, and has been clinically recognized in recent years (Groskopf et al., 2006; Lemos et al., 2019). Not only in urine, but also in gastric juice, lncRNAs have been suggested as a marker for digestive tumors (Shao et al., 2016). We note that there are relevant experiments investigating the prognostic effectiveness of copper ion carrier small molecule anticancer drugs in patients with lung adenocarcinoma (Soma et al., 2018). In addition, a new study has constructed a prognostic model for ccRCC associated with cuproptosis, and it is undeniable that the results of this study have some significance for the study of cuproptosis in ccRCC (Bian et al., 2022), but lncRNA, as an important part of liquid biopsy, is important to study the potential value of Cuproptosis-related lncRNAs in ccRCC.

The cuproptosis-related lncRNAs in this study were obtained by co-expression of cuproptosis-related genes and lncRNAs, and a predictive model for ccRCC prognosis was constructed using five cuproptosis-related lncRNAs (FOXD2-AS1, SUCLG2-AS1, LINC00271, NUP153-AS1, and LINC02154). Risk scores were obtained as independent prognostic factors for ccRCC patients by univariable and multivariable Cox analyses, and we likewise found that the HR of risk score was only slightly higher than 1.0 in both univariable and multivariable Cox analyses. The vast majority of ccRCC prognostic models already published have a risk score HR slightly higher than 1.0. Therefore, we consider this to be a generalization of the ccRCC prognostic model. However, since its lowest value is still greater than 1.0, the risk score is a statistically significant independent prognostic factor for ccRCC patients (Han et al., 2020; Chang et al., 2021; Li et al., 2021). The validation of the internal cohort and external cohort showed that the model has a good ability to predict the prognosis of patients with ccRCC. We found AUC values of 0.768, 0.757, and 0.773 for the 1-year, 3-years, and 5-years ROC curves, respectively, of 431 ccRCC patients with clinical data on DFS. Compared with AUCs of 0.622, 0.634, and 0.682 for the 1-year, 3-years, and 5-years ROC curves, respectively, from Bian et al. (2022) for predicted DFS, the AUC values of this study were generally higher than those of Bian’s research team. However, we believe that they are complementary at the study level and mutually corroborate that the models developed by both teams highlight the potential value of cuproptosis for the study of ccRCC. To investigate the TME characteristics of the high- and hypo-risk categories, we performed immune cell analysis, TME scoring, and immune checkpoint analysis for the high- and hypo-risk categories using different algorithms. The majority of immune checkpoints were expressed at high levels in the hyper-risk category, but PD-L1 checkpoint was at high level in the hypo-risk category, indicating that anti-PD-L1 therapy may be more effective in the hypo-risk category. In addition, we analyzed the clinical correlation and stratification of high- and hypo-risk categories, and the results demonstrated significant differences in clinical characteristics and stratification between high- and hypo-risk categories, apart from the fact that the model score was also an independent prognostic element. In order to improve the predictive effect that the model has on clinical medication usage, we found that patients from the hypo-risk category were considered more susceptible to most drugs than those from the hypo-risk category. This model will help researchers figure out how cuproptosis-related lncRNAs might affect the prognosis of patients with ccRCC, and come up with new ways to treat ccRCC.

Multiple reports have shown that FOXD2-AS1 plays an important role in the development of multiple tumors, including the promotion of malignant progression of bile duct cancer via regulation of the miR-760/E2F3 axis (Hu et al., 2022). FOXD2-AS1 may promote pancreatic cancer cell invasion and migration by sponging miR-30a-3p, which upregulates COX-2 (Ye et al., 2022). FOXD2-AS1 promotes breast cancer cell proliferation, invasion, migration, and drug resistance by positively regulating the PI3K/AKT signaling pathway, inhibits apoptosis, and accelerates breast cancer progression (Nong et al., 2021). We discovered that no relevant literature has reported its role in ccRCC. FOXD2-AS1 was found to be more expressed in the hyper-risk category with a short survival time than in the hypo-risk category with a long survival time in this study, suggesting that FOXD2-AS1 may be a promoter for ccRCC, and opening up possibilities for future research. SUCLG2-AS1 has been proposed as a prognostic marker for triple-negative breast cancer and ccRCC, despite the fact that the mechanism by which it affects tumor development remains unknown (Wu et al., 2021; Yang et al., 2021). Consistent with previously reported findings, our findings suggest that it is enriched in the hypo-risk category with long survival time, and has a possible inhibitory effect on tumor development. In 2016, Ma et al. (2016) identified LINC00271 as an independent risk factor for the recurrence of papillary thyroid cancer, observing that its expression was downregulated in numerous tumors, including breast cancer, renal medullary cell carcinoma, and head-and-neck squamous cell carcinoma. Our study found that its expression was enriched in the low-risk group, similar to the study by Ma et al., implying a negative association between LINC00271 and the development of ccRCC. Yue et al. discovered that LINC02154 promotes hepatocellular carcinoma proliferation and metastasis, by increasing SPC24 promoter activity and activating the PI3K-AKT signaling pathway (Yue et al., 2022). LINC02154 is a cancer-promoting factor not only in liver cancer but also in laryngeal squamous cell carcinoma (Zhang et al., 2019). Consistent with previous findings, the present study showed that LINC02154 was expressed at a high level in the hyper-risk category with a poor prognosis. We found no reports regarding NUP153-AS1 in the relevant literature, but the results of this study showed that it was enriched in the hypo-risk category with a long survival time, suggesting that NUP153-AS1 may play an oncogenic role in the progression of ccRCC, and provide options for future researchers.

Surgical resection remains the main treatment for ccRCC as one of the most common urological tumors, but not all ccRCC patients tolerate surgery. Thus, new treatment options are needed. Immunotherapy, a relatively new treatment, has been shown to have significant therapeutic value in a number of tumors (Shibata et al., 2019; Zhou et al., 2019). We assessed the immune cell infiltration in the high- and hypo-risk categories using different platforms, and showed that cancer-associated fibroblasts, M0 macrophages, M1 macrophages, monocytes, NK cells, CD4^+^ memory activated T cells, CD8^+^ T cells, and regulatory T cells (Tregs) were infiltrated to a higher extent in the high-risk than in the hypo-risk category, while M2 macrophages, B cells, neutrophils, and mast cells were infiltrated to a higher extent in the hypo-risk than in the hyper-risk category. M1 macrophages and M2 macrophages are the two primary phenotypes of tumor-associated macrophages. M1 macrophages serve as antitumor cells, by producing pro-inflammatory type I cytokines (Pan et al., 2020). We discovered a higher degree of M1 macrophage infiltration in the hyper-risk category compared to the hypo-risk category, indicating that immunotherapy may be advantageous for the hyper-risk category. Despite the fact that M2 macrophages have immunosuppressive properties and promote tumor development, our findings of greater M2 macrophage infiltration in the hypo-risk category suggest that the hypo-risk category may respond less favorably to immunotherapy than the hyper-risk category.

With the expansion of immunotherapy research, improved individualization of treatment is also a current hot topic. We divided ccRCC patients into high- and low-risk groups, as well as C1 and C2 subtypes, based on the five lncRNAs mentioned above. We found that there was some overlap between the high- and low-risk groups and the different subtypes, which may represent a convergence in prognosis and immune infiltration between them. This was subsequently demonstrated by analyzing the TME scores of different risk groups and subtypes, which were significantly higher in the high-risk group and C2 subtype than in the low-risk group and C1 subtype, and in the subsequent analysis of immune checkpoint activation, we found that the majority of immune checkpoints were upregulated in the high-risk group and C2 subtype. We also discovered that the PDL-1 checkpoints were reversed, implying that patients with hyper-risk ccRCC and the C2 subtype may benefit from immunotherapy, while those with hypo-risk ccRCC and the C1 subtype may be more sensitive to anti-PDL-1. These findings have significant implications for the development of personalized immunotherapy for patients with ccRCC.

Although we have applied many methods to enrich our model, it still has some shortcomings. The model is built based on the TCGA database. We have validated the accuracy of the model by an internal validation set, as well as an external validation set, and the immune cell infiltration analysis by different platforms can be used as an external validation in a sense. However, we have not further investigated and validated the role of these cuproptosis-related lncRNAs in ccRCC tumorigenesis and tumor progression through experimental studies (Hong et al., 2020; Xu et al., 2021). Even with these limitations, our study has some potential implications for patient survival prediction, and individualized immunotherapy. In the future, we will enrich and confirm the value of cuproptosis-related lncRNAs by collecting more clinical datasets and through experimental studies.

## Conclusion

From our research, we identified five cuproptosis-related lncRNAs with prognostic values and used them to develop a model to anticipate the prognosis of ccRCC patients, which provides an important basis for individualized treatment of ccRCC patients. In the future, targeting cuproptosis-associated lncRNAs will be a promising therapeutic strategy for ccRCC patients.

## Data Availability

Publicly available datasets were analyzed in this study. This data can be found here: https://portal.gdc.cancer.gov/.
