# Supplementary material for: Cuproptosis-related lncRNA: Prediction of prognosis and subtype determination in clear cell renal cell carcinoma
Source: Front Genet. 2022 Aug 22;13:958547. doi: 10.3389/fgene.2022.958547 (PMC9441767; doi:10.3389/fgene.2022.958547)
Supplement: Supplementary file 1 [file DataSheet1.ZIP › Supplementary Figures.docx]

**Supplementary Figure**


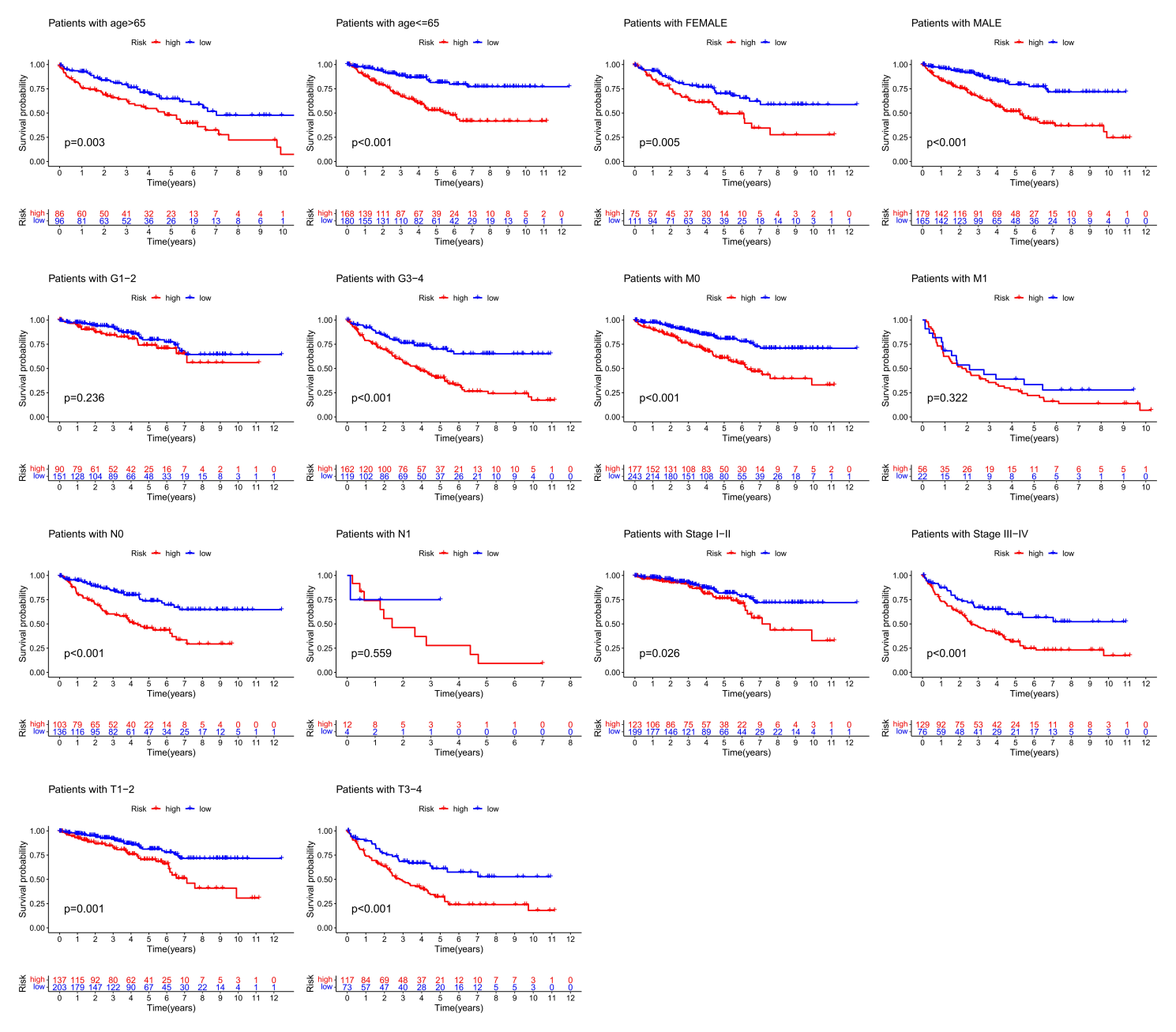


**(Supplementary Figure 1:**Correlation analysis of high and low risk groups in different clinicopathological characteristics.)


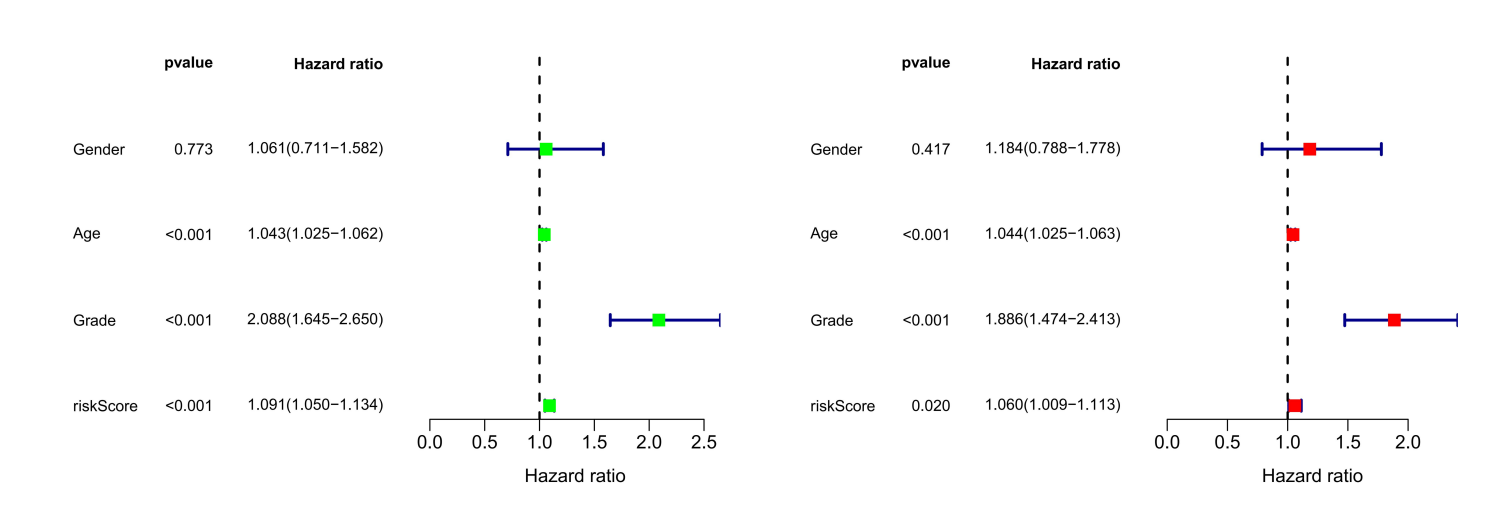


**(Supplementary Figure 2:**univariate and multifactorial COX analysis of combined data from the beta group and the E-MTAB-1980 cohort.)


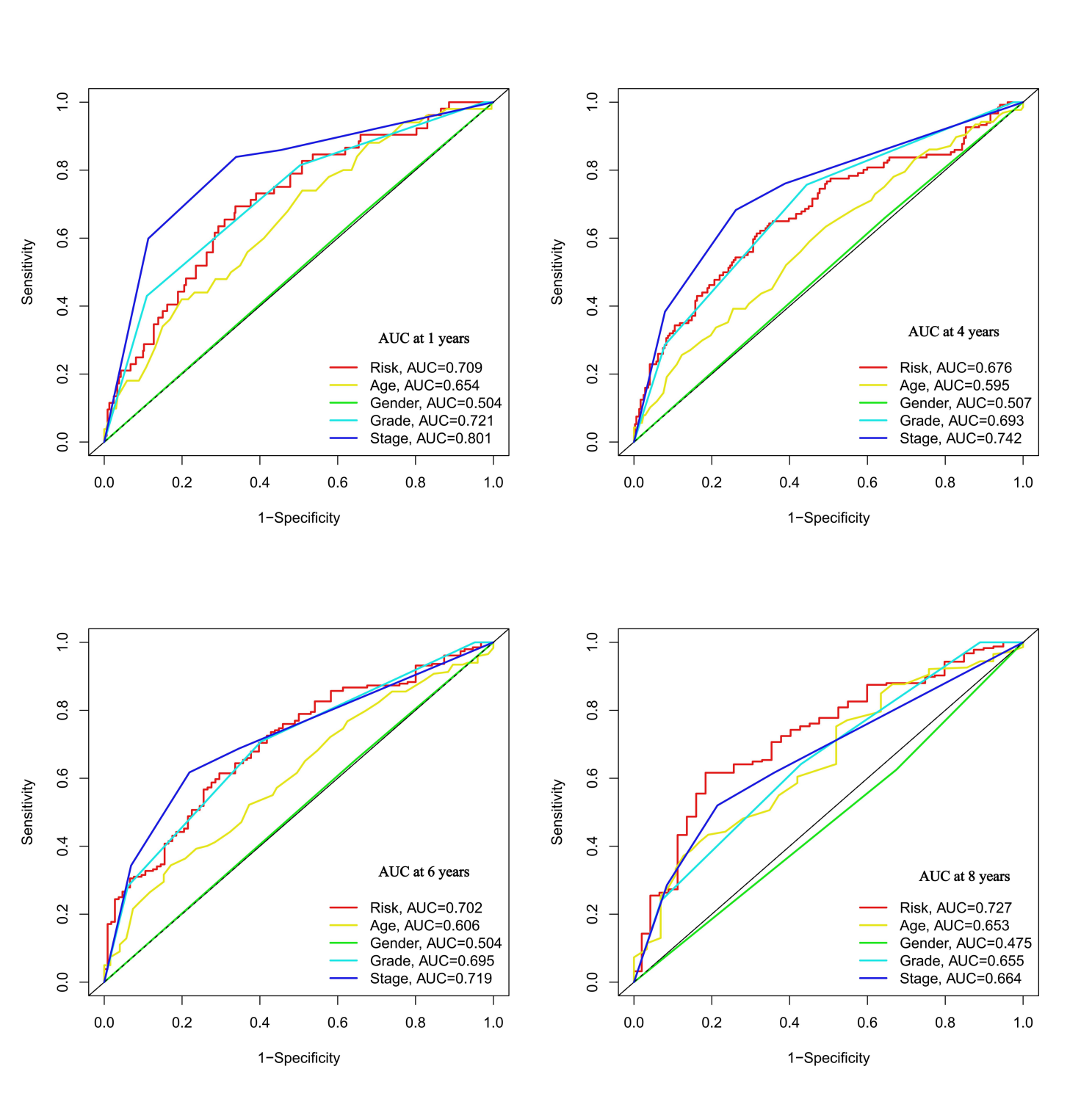


**(Supplementary Figure 3:**Clinical characteristics and risk scores 1, 4, 6, and 8 year ROC curves.)


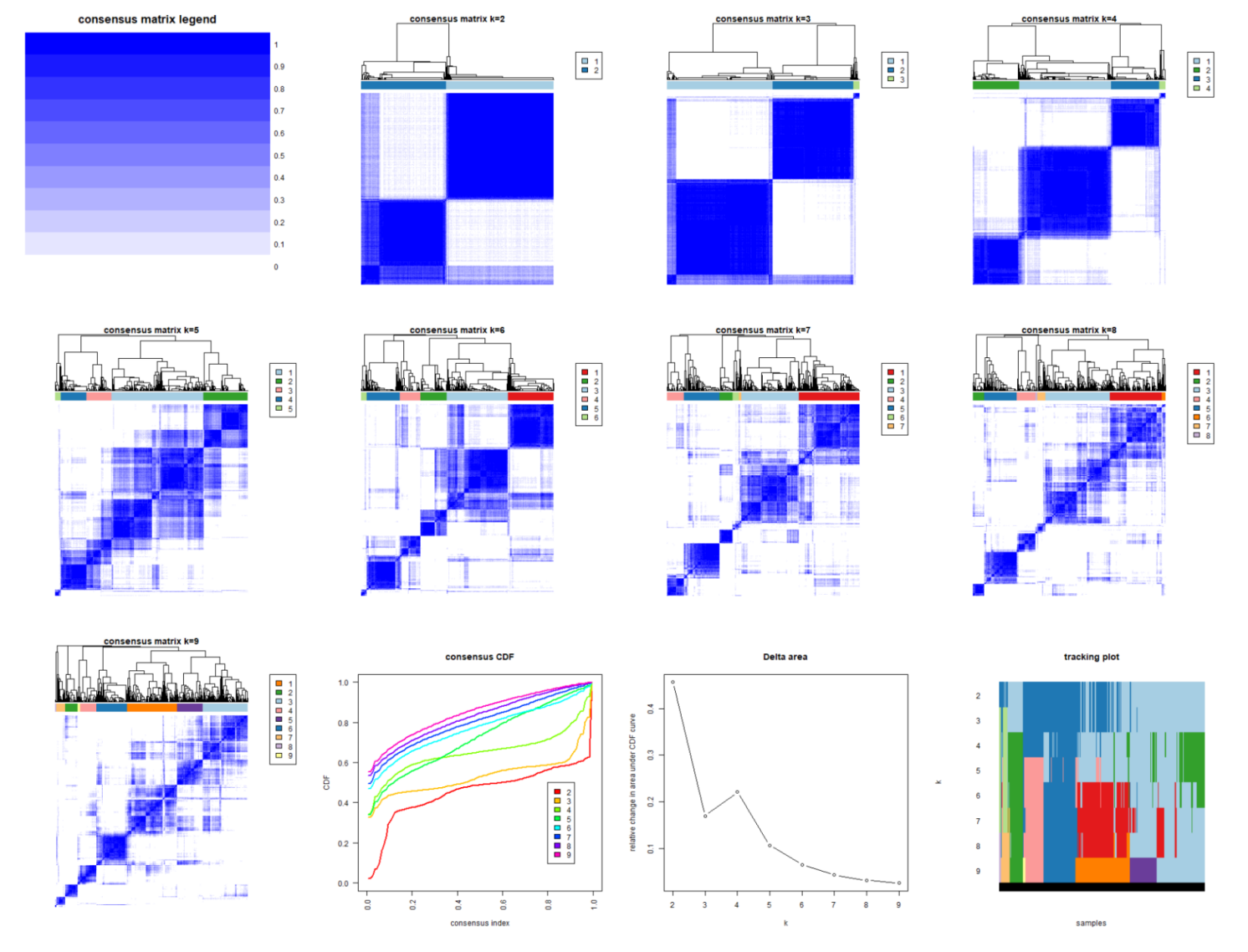


**(Supplementary Figure 4:**Consistent class aggregation results for k=1 to k=9.)
